# Psychiatric Documentation and Management in Primary Care With Artificial Intelligence Scribe Use

**DOI:** 10.1001/jamapsychiatry.2025.4303

**Published:** 2026-01-21

**Authors:** Victor M. Castro, Thomas H. McCoy, Pilar Verhaak, Anudeepa Ramachandiran, Roy H. Perlis

**Affiliations:** 1 Center for Quantitative Health and Department of Psychiatry, Massachusetts General Hospital, Boston; 2 Department of Psychiatry, Harvard Medical School, Boston, Massachusetts

## Abstract

**Importance:**

Despite increasingly widespread use of artificial intelligence (AI)–driven ambient scribes in medicine, the extent to which they are associated with clinician practice is not well studied.

**Objective:**

To characterize differences in documentation and treatment of psychiatric symptoms in primary care outpatient notes generated using ambient scribes compared with human or no scribes.

**Design, Setting, and Participants:**

This cohort study used a matched retrospective case-control design to evaluate primary care annual visit notes from the Massachusetts General and Brigham and Women’s Hospital systems between February 2023 and February 2025. A random sample of notes from 4 types of visits, matched 1:1 using sociodemographic and clinical features, was used: those using an ambient scribe, those using a human scribe, those occurring during the same period without a scribe (contemporaneous), and those occurring prior to scribe deployment. Data analysis was performed from April 25 to May 1, 2025.

**Exposure:**

Use of an AI ambient scribe.

**Main Outcomes and Measures:**

Neuropsychiatric symptom documentation, in terms of estimated Research Domain Criteria (RDoC), using a Health Insurance Portability and Accountability Act–compliant large language model (GPT-4o version gpt-4o-11-20; OpenAI); antidepressant prescriptions and diagnostic codes; and referral for mental health follow-up.

**Results:**

Among 20 302 notes, the mean (SD) age of the patients was 48 (14) years and 11 960 (59%) were for visits by female patients; 1026 (5%) met criteria for moderate or greater depressive symptoms by Patient Health Questionnaire-9 score. Estimated levels of RDoC symptoms in all 6 domains were significantly greater in the AI-scribed notes compared with other groups. In a multiple logistic regression model, likelihood of a psychiatric intervention (referral, new diagnosis, or antidepressant prescription) was significantly lower among AI-scribed visits compared with contemporaneous unscribed visits (adjusted odds ratio, 0.83; 95% CI, 0.72-0.95), but not for human-scribed visits compared with contemporaneous unscribed visits (adjusted odds ratio, 0.97; 95% CI, 0.85-1.11).

**Conclusions and Relevance:**

In this retrospective cohort study using a matched case-control design examining outpatient primary care notes, incorporation of AI ambient scribes in primary care was associated with greater levels of neuropsychiatric symptom documentation but lesser likelihood of documented management of psychiatric symptoms. Further study will be required to determine whether these changes are associated with differential outcomes.

## Key Points

**Question:**

How are documentation and management of psychiatric symptoms in primary care outpatient visits different using artificial intelligence–driven ambient scribes vs human or no scribes?

**Findings:**

In this cohort study of more than 20 000 routine annual visits, ambient scribe use was associated with modestly greater documentation of neuropsychiatric symptoms but less likelihood of a depression-related intervention or diagnostic code.

**Meaning:**

The extent to which use of ambient scribes may be associated with altered response to psychiatric symptoms by clinicians merits further investigation.

## Teaser

This cohort study using a matched case-control design characterizes differences in documentation and treatment of psychiatric symptoms in primary care outpatient notes generated using ambient scribes compared with human or no scribes.

## Introduction

The use of artificial intelligence (AI) ambient scribes, applying speech recognition and large language models to automate narrative note generation, has rapidly become widespread in medicine. To date, investigations of the impact of this transition to AI scribes are limited. One study suggested that clinicians using ambient scribes spent, on average, 5 minutes less per visit using the electronic health record^1^; others yielded mixed results,^2,3,4^ with one study suggesting little change in measures of clinician productivity.^5^ A broader set of studies have suggested that efforts to improve documentation in electronic health records correlate with improvements in overall quality of patient care.^6,7^

In particular, little is known about how the use of ambient scribes may change documentation and management of neuropsychiatric symptoms. Despite efforts to encourage more systematic symptom measurement for depression,^8,9^ prior work suggested that incorporation of the Patient Health Questionnaire-9 (PHQ-9) as a patient-reported outcome was associated with diminished documentation of depressive symptoms.^10^ It is possible that use of scribes could ameliorate this diminution, providing clinicians more time to discuss mental health with patients at annual visits and increasing the likelihood that such symptoms are documented. On the other hand, if mental health is not prioritized in the visit, no such shift would be observed. While prior findings could reflect a lack of documentation but not discussion, a lack of change here might indicate a lack of discussion.

To address this gap in knowledge, we drew on the electronic health records of annual visits in outpatient primary care clinics of 2 large academic health systems in eastern Massachusetts, which span academic medical centers, community hospitals, and affiliated outpatient practices. During the period examined, ambient AI scribing was gradually deployed across the system in a nonuniform fashion. We identified contemporaneous groups (ie, from practices or clinicians not yet transitioned to AI scribes, and practices using non-AI human scribes), plus an additional set of notes from a prior year, for comparison with notes generated via AI scribing. We hypothesized that, with the shift to AI scribes, documentation of neuropsychiatric symptoms would be greater than without scribes, with a concomitant increase in intervention for such symptoms.

## Methods

### Study Design and Sample Derivation

This cohort study used a matched retrospective case-control design. From the electronic health records of 2 large academic medical centers in Eastern Massachusetts, we drew a random sample of 5076 outpatient primary care annual visit notes, identified by a corresponding *Current Procedural Terminology* code (99385-99387, 99395-99397) or Healthcare Common Procedure Coding System code (G0402, G0438, G0439), among individuals aged 18 years or older seen between February 2024 and February 2025. We included those who had an associated PHQ-9 score recorded and whose note included documentation of AI scribe use. Such use was determined based on documentation of patient consent to use of the technology in the clinical note. For comparison, we drew 3 additional samples of outpatient annual visit notes matched 1:1 on age at visit, sex, race (self-reported as recorded in the electronic health record, included here as a means of controlling for differences in clinic populations), and prior depressive disorder diagnosis (*International Statistical Classification of Diseases and Related Health Problems, Tenth Revision* [*ICD-10*] codes F06.3*, F32.*, F33.*, F34.1, and F53.0): one including documentation of a human scribe (identified using standard text in the clinical note), one including no such documentation between February 2024 and February 2025, and one with no such documentation from a period before ambient scribe use, between February 2023 and January 2024 (the prior-year sample).

A total of 20 302 notes across 4 types of visits were included in the analysis. For each visit, we identified sociodemographic features, including sex, age at visit, self-reported race and ethnicity, highest level of education, and insurance status (commercial or otherwise), to facilitate comparisons of the cohorts. Additionally, we identified *ICD-10* codes corresponding to the visit, RxNorm codes indicating the prescription of an antidepressant (including selective serotonin reuptake inhibitors, serotonin–norepinephrine reuptake inhibitors, tricyclic antidepressants, bupropion, and trazodone), clinician orders for ambulatory referrals for psychiatric evaluation, and corresponding PHQ-9 scores.

The study was approved by the institutional review board of Mass General Brigham, which granted a waiver of informed consent as it would be infeasible to contact participants and no participant interaction was required. Data analysis was performed from April 25 to May 1, 2025.

### Generation of Estimated Research Domain Criteria Scores

In prior work, we demonstrated that language models can readily estimate dimensions of National Institute of Mental Health Research Domain Criteria (RDoC)^11^ symptoms.^12^ We applied this method to characterize such scores in each narrative clinical note. Specifically, we applied a Python script to present individual narrative clinical notes via an application programming interface to a Health Insurance Portability and Accountability Act–compliant instance of GPT4o (version gpt-4o-11-20; OpenAI) hosted by Microsoft Azure, with model temperature set at 0 to yield results as deterministic as possible. The prompt was, “You are a skilled psychiatrist scoring an outpatient visit note in terms of how the patient symptoms over the past 24 hours reflect the 6 RDoC domains: Negative Valence Systems, Positive Valence Systems, Cognitive Systems, Social Processes, Arousal and Regulatory Systems, and Sensorimotor Systems. Remember that substance use can be reflected as a Positive Valence symptom. Notes are scored on a 0-10 scale to capture the magnitude of documented symptoms relevant in a given domain. Score 0 if no symptoms are present and functioning is normal, 1-3 mild symptoms, 4-6 moderate, 7-9 severe, 10 extremely severe. Generally, moderate requires treatment and severe requires hospitalization. Return only a JSON [JavaScript Object Notation] object with each score, Negative, Positive, Cognitive, Social, Arousal, and Sensorimotor.” The model was reinitialized after each presentation to ensure prior notes could not influence subsequent scores.

### Statistical Analysis

In univariate analyses, we compared features of ambient AI-scribed notes vs those of the comparator groups using Mann-Whitney test or χ^2^ test. We similarly compared estimated RDoC domain scores. Primary analyses focused on comparing AI-scribed vs human-scribed notes and vs contemporaneous unscribed notes; we also included a prior unscribed set (ie, before AI scribes were deployed) to exclude the possibility that contemporaneous unscribed notes reflected other differences. We examined an aggregate visit outcome of depression diagnosis, new antidepressant prescription, or referral for psychiatric evaluation and care—the last of these does not record a specific indication for referral and thus is broader than depression evaluation per se—and, separately, new antidepressant prescription. Beyond univariate contrasts, we fit multiple logistic regression models to examine associations between scribe type and likelihood of our aggregate outcome, adjusted for sociodemographic features.

A nominal *P* value of .05, 2-tailed, was set as the threshold for statistical significance. Bonferroni correction for note length, 6 RDoC domains, 2 additional measures, and the composite outcome would require *P* < .005 for statistical significance. All analyses used R version 4.4.0 statistical software (R Foundation).^13^

## Results

Characteristics of individuals reflected in the visit notes from each of the 4 groups are shown in Table 1. The mean (SD) age at visit was 48 (14) years in all groups; 11 960 notes (59%) were for visits by female patients. Mean (SD) PHQ-9 scores were similar across groups: 1.2 (3.5) for ambient AI scribe, 1.2 (3.6) for human virtual scribe, 1.2 (3.5) for no scribe (after deployment), and 1.2 (3.6) for no scribe (prior to ambient AI scribe technology deployment). The proportion of visits reflecting a PHQ-9 score greater than or equal to 10 was 1026 (5.0%) overall and was similar across groups: 254 (5%) for ambient AI scribe, 256 (5%) for human virtual scribe, 265 (5%) for no scribe (after deployment), and 251 (5%) for no scribe (prior to deployment).

**Table 1.** Characteristics of Individuals in the Ambient AI Scribe and Comparator Cohorts

Characteristic	Ambient AI scribe (n = 5076)	Human virtual scribe (n = 5075)	No scribe	
			After deployment (n = 5075)^a^	Prior to deployment (n = 5076)^b^
Age at visit, mean (SD), y	48 (14)	48 (14)	48 (14)	48 (14)
Sex, No. (%)				
Female	2989 (59)	3002 (59)	2981 (59)	2988 (59)
Male	2087 (41)	2073 (41)	2094 (41)	2088 (41)
Race, No. (%)^c^				
Asian	567 (11)	574 (11)	566 (11)	567 (11)
Black	344 (7)	320 (6)	351 (7)	318 (6)
White	3601 (71)	3620 (71)	3598 (71)	3601 (71)
Other^d^	564 (11)	561 (11)	560 (11)	590 (12)
Ethnicity, No. (%)^c^				
Hispanic	519 (10)	576 (11)	500 (10)	527 (10)
Non-Hispanic	4557 (90)	4499 (89)	4575 (90)	4549 (90)
At least bachelor’s degree, No. (%)	3035 (60)	2948 (58)	2914 (57)^e^	2851 (56)^f^
Public insurance, No. (%)	250 (5)	172 (3)^g^	199 (4)^e^	224 (4)
*ICD-10* depression code in prior 2 y, No. (%)	867 (17)	991 (20)^f^	996 (20)^f^	955 (19)^e^
PHQ-9 score				
Mean (SD)	1.2 (3.5)	1.2 (3.6)	1.2 (3.5)	1.2 (3.6)
≥10, No. (%)	254 (5)	256 (5)	265 (5)	251 (5)

Abbreviations: AI, artificial intelligence; *ICD-10*, *International Statistical Classification of Diseases and Related Health Problems, Tenth Revision*; PHQ-9, Patient Health Questionnaire-9.

^a^ Notes written after the deployment of ambient AI scribe technology.

^b^ Notes written prior to the deployment of ambient AI scribe technology.

^c^ Race and ethnicity were self-reported as recorded in the electronic health record and is included here as a means of controlling for differences in clinic populations.

^d^ Includes American Indian or Alaska Native, Native Hawaiian or Other Pacific Islander, multiple races, or unknown; these were collapsed into a single group owing to small sample sizes.

^e^ Bonferroni-corrected *P* < .05 vs ambient AI scribe group via χ^2^ test.

^f^ Bonferroni-corrected *P* < .01 vs ambient AI scribe group via χ^2^ test.

^g^ Bonferroni-corrected *P* < .001 vs ambient AI scribe group via χ^2^ test.

**Figure 1.**
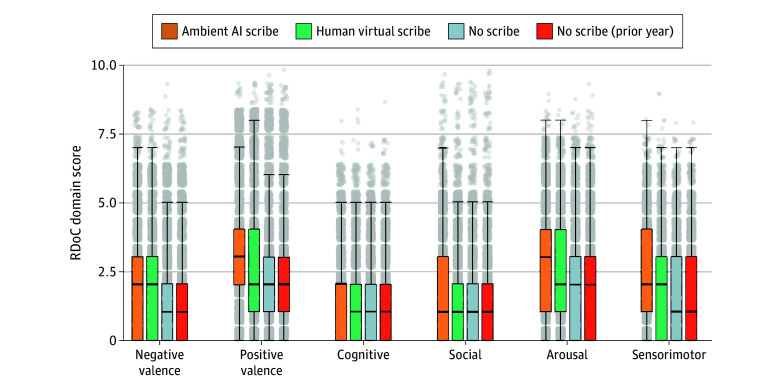
Estimates of Research Domain Criteria (RDoC) Symptoms by Visit Type AI indicates artificial intelligence; center horizontal lines, median; bottom and top borders of boxes, 25th and 75th percentile, respectively; whiskers, 1.5 times the IQR.

We first compared overall note length between groups. Length was greatest in the human virtual scribe group, with a mean (SD) of 16 252 (7060) characters, followed by ambient AI scribe at 13 629 (4507) characters, no scribe (after deployment) at 7932 (5025) characters, and no scribe (prior to deployment) at 7489 (4738) characters (*P* < .001 for all contrasts). We then examined RDoC domain scores estimated from the note text. Across all 6 domains, scores were significantly higher in the ambient AI scribe group compared with the other groups (Figure 1 and Table 2). For negative valence, mean (SD) scores were 2.05 (1.82) with ambient AI scribe, 1.79 (1.77) with human virtual scribe, 1.57 (1.77) with no scribe (after deployment), and 1.55 (1.75) with no scribe (prior to deployment). Mean (SD) positive valence scores were 3.14 (2.22), 2.74 (2.19), 2.34 (1.99), and 2.29 (1.98), respectively; mean (SD) cognitive domain scores were 1.66 (1.48), 1.37 (1.49), 1.27 (1.48), and 1.26 (1.49), respectively; mean (SD) social domain scores were 1.70 (1.70), 1.46 (1.63), 1.28 (1.57), and 1.26 (1.57), respectively; mean (SD) arousal scores were 2.84 (1.83), 2.43 (1.83), 2.05 (1.85), and 2.03 (1.83), respectively; and mean (SD) sensorimotor scores were 2.33 (1.70), 1.77 (1.62), 1.54 (1.59), and 1.53 (1.60), respectively. All contrasts were statistically significant at *P* < .001.

**Table 2.** Comparison of Note Length and Large Language Model–Computed RDoC Domain Scores in Note Text

Characteristic	Mean (SD)			
	Ambient AI scribe (n = 5076)	Human virtual scribe (n = 5075)	No scribe	
			After deployment (n = 5075)^a^	Prior to deployment (n = 5076)^b^
Note length, No. of characters	13 629 (4507)	16 252 (7060)^c^	7932 (5025)^c^	7489 (4738)^c^
RDoC domain score				
Negative valence	2.05 (1.82)	1.79 (1.77)^c^	1.57 (1.77)^c^	1.55 (1.75)^c^
Positive valence	3.14 (2.22)	2.74 (2.19)^c^	2.34 (1.99)^c^	2.29 (1.98)^c^
Cognitive	1.66 (1.48)	1.37 (1.49)^c^	1.27 (1.48)^c^	1.26 (1.49)^c^
Social	1.70 (1.70)	1.46 (1.63)^c^	1.28 (1.57)^c^	1.26 (1.57)^c^
Arousal	2.84 (1.83)	2.43 (1.83)^c^	2.05 (1.85)^c^	2.03 (1.83)^c^
Sensorimotor	2.33 (1.70)	1.77 (1.62)^c^	1.54 (1.59)^c^	1.53 (1.60)^c^

Abbreviations: AI, artificial intelligence; RDoC, Research Domain Criteria.

^a^ Notes written after the deployment of ambient AI scribe technology.

^b^ Notes written prior to the deployment of ambient AI scribe technology.

^c^ Bonferroni-corrected *P* < .001 vs ambient AI scribe group via Mann-Whitney test.

Visits were associated with a depression-related *ICD-10* code in 447 (9%) with ambient AI scribe, 587 (12%) with human virtual scribe (Bonferroni-corrected *P* < .001 vs ambient AI scribe), 604 (12%) with no scribe (after deployment; Bonferroni-corrected *P* < .001 vs ambient AI scribe), and 535 (11%) with no scribe (prior to deployment; Bonferroni-corrected *P* < .01 vs ambient AI scribe) (Table 3). Antidepressants were prescribed in 275 visits (5%) with ambient AI scribe, 313 (6%) with human virtual scribe, 317 (6%) with no scribe (after deployment), and 319 (6%) with no scribe (prior to deployment); none of these contrasts were statistically significant. Similarly, a behavioral health referral was entered in 151 visits (3%), 133 (3%), 150 (3%), and 113 (2%), respectively; the differences were not statistically significant. Finally, the composite depression outcome—defined as the presence of any of diagnosis, antidepressant prescription, or behavioral health referral—was lower in the ambient AI scribe group at 708 visits (14%) compared with 843 visits (17%) with human virtual scribe (Bonferroni-corrected *P* < .001 vs ambient AI scribe), 855 (17%) with no scribe (after deployment; Bonferroni-corrected *P* < .001 vs ambient AI scribe), and 805 (16%) with no scribe (prior to deployment; *P* < .05 vs ambient AI scribe).

**Table 3.** Comparison of Depression Interventions at Annual Visit

Characteristic	No. (%)			
	Ambient AI scribe (n = 5076)	Human virtual scribe (n = 5075)	No scribe	
			After deployment (n = 5075)^a^	Prior to deployment (n = 5076)^b^
*ICD-10* depression code at visit	447 (9)	587 (12)^c^	604 (12)^c^	535 (11)^d^
Antidepressant prescription	275 (5)	313 (6)	317 (6)	319 (6)
New antidepressant prescription	52 (1)	50 (1)	66 (1)	88 (2)^d^
Referral with order for behavioral health	151 (3)	133 (3)	150 (3)	113 (2)
Composite depression outcome	708 (14)	843 (17)^c^	855 (17)^c^	805 (16)^e^

Abbreviations: AI, artificial intelligence; *ICD-10*, *International Statistical Classification of Diseases and Related Health Problems, Tenth Revision*.

^a^ Notes written after the deployment of ambient AI scribe technology.

^b^ Notes written prior to the deployment of ambient AI scribe technology.

^c^ Bonferroni-corrected *P* < .001 vs ambient AI scribe group via χ^2^ test.

^d^ Bonferroni-corrected *P* < .01 vs ambient AI scribe group via χ^2^ test.

^e^ Bonferroni-corrected *P* < .05 vs ambient AI scribe group via χ^2^ test.

**Figure 2.**
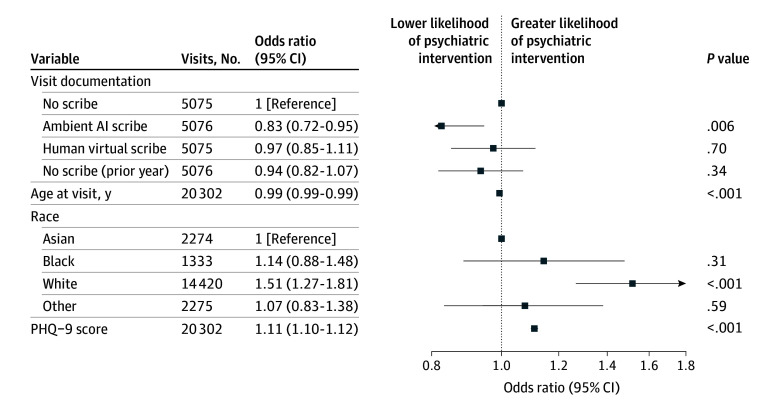
Multiple Logistic Regression Model of Psychiatric Intervention at Visit Race was self-reported as recorded in the electronic health record. Other race includes American Indian or Alaska Native, Native Hawaiian or Other Pacific Islander, multiple races, or unknown; these were collapsed into a single group owing to small sample sizes. AI indicates artificial intelligence; PHQ-9, Patient Health Questionnaire-9.

While the groups were matched on age, sex, and race and ethnicity, we next examined whether scribe use was associated with these differential outcomes after accounting for sociodemographic and clinical features. In a multiple logistic regression model (Figure 2), likelihood of any psychiatric intervention was significantly lower among AI-scribed visits compared with unscribed visits (after deployment) (adjusted odds ratio [aOR], 0.83; 95% CI, 0.72-0.95); no difference was observed between human-scribed visits (aOR, 0.97; 95% CI, 0.85-1.11) or unscribed visits (prior to deployment) (aOR, 0.94; 95% CI, 0.82-1.07) compared with unscribed visits (after deployment). (Prior depression diagnosis is omitted from the figure to allow other outcomes to be more readily inspected; the aOR was 21.30 [95% CI, 19.32-23.50].)

## Discussion

In this study examining clinical documentation from more than 20 000 outpatient annual visits, including roughly 5000 incorporating AI scribes, we found that use of these scribes was associated with greater documented levels of neuropsychiatric symptoms compared with the use of human scribes or no scribe but lesser likelihood of a depression intervention.

Some^2,3,4^ but not all^5^ studies have suggested that ambient AI scribes may reduce clinician workload, with concomitant improvement in clinician satisfaction,^3,4,14^ but we could not identify comparable studies examining documentation itself. Other efforts to structure and standardize clinical documentation have generally been associated with improved note quality.^6,7^ Conversely, one randomized clinical trial of templated notes found that, while organization of such notes improved, they were felt to be less useful and to have lower accuracy when evaluated by clinical faculty.^15^ More generally, the potential adverse outcomes of language models on the reliability of clinical documentation was suggested by a recent commentary.^16^ In this regard, our results are reassuring, suggesting that AI scribes in primary care have the potential to increase documentation of neuropsychiatric symptoms.

On the other hand, this increased documentation did not translate to increased attention to depressive symptoms: visits incorporating AI scribes were actually less likely to be associated with psychiatric interventions. One explanation for this association could be that automating documentation leads clinicians to be less active in general, analogous to reduced proficiency observed in pilots after the emergence of autopilot.^17^ While this effect was not observed in human-scribed visits, it is possible that clinician attentiveness varies depending on the nature of the scribe.

In general, the rapid dissemination of AI scribes in medicine poses both an opportunity and a risk. Our results hint at the opportunity, if greater documentation of symptoms equates to greater response to those symptoms. On the other hand, many interventions in medicine have been adopted without clear evidence of benefit—particularly those, like scribes, that do not require formal regulatory review to establish effectiveness. To date, most investigations of AI scribes have focused on impact on health care professionals but less so on patient care. Prospective studies will be valuable in understanding the potential for scribes to improve care and in understanding their liabilities. Our work suggests that scribes do change documentation substantially, but the impact on care quality remains to be determined.

### Limitations

This study has multiple limitations. While we generated multiple comparison groups—contemporaneous, prior to deployment of ambient AI scribes, or contemporaneous with use of human scribes—we cannot exclude the possibility that associations between scribe use and measured note characteristics are confounded in other ways. The rapid dissemination of ambient AI scribes may largely preclude randomized clinical trials, but at minimum, larger-scale prospective studies may be needed to determine causal effects. For example, differences in patient populations not captured by sociodemographic features could account for differential documentation. Alternatively, differences in health care professionals’ acceptance of ambient scribes, or access to such scribes, could confound results. Such health care professional–level data were not available to us but would be valuable to consider in future work. Additionally, all notes reflect the use of ambient AI scribes in primary care in a single geographic region among affiliated health systems, albeit in varied clinics across the region. This is a potential strength in reducing overall practice heterogeneity, but whether these outcomes apply in other populations or generalize to all ambient AI scribe systems remains to be investigated.

## Conclusions

While ambient AI scribes have rapidly become a standard approach to clinical documentation, this cohort study of more than 20 000 outpatient annual visit notes suggests that visits using AI scribes may differ in clinically meaningful ways from those that rely on human scribes or do not use scribes at all. This work suggests that further investigation will be critical for understanding the potential for AI scribe use, and application of language models for documentation more generally, to impact neuropsychiatric symptom documentation in primary care and for understanding potential consequences.

## Notes

Supplement. Data Sharing Statement
