# Supplementary material for: Psychiatric Documentation and Management in Primary Care With Artificial Intelligence Scribe Use
Source: JAMA Psychiatry. 2026 Jan 21;83(3):281–6. doi: 10.1001/jamapsychiatry.2025.4303 (PMC12824846; doi:10.1001/jamapsychiatry.2025.4303)
Supplement: Supplement. — Data Sharing Statement [file jamapsychiatry-e254303-s001.pdf]

# Data Sharing Statement

Castro. Psychiatric Documentation and Management in Primary Care With Artificial Intelligence Scribe Use. *JAMA Psychiatry*. Published January 21, 2026.
doi:10.1001/jamapsychiatry.2025.4303

**Data**
**Data available:** No
